# Percutaneous closure of accidentally subclavian artery catheterization: time to change first line approach?

**DOI:** 10.1186/s42155-022-00300-7

**Published:** 2022-05-25

**Authors:** Andrea Discalzi, Claudio Maglia, Fernanda Ciferri, Andrea Mancini, Lorenzo Gibello, Marco Calandri, Gianfranco Varetto, Paolo Fonio

**Affiliations:** 1 Department of Surgical Sciences Radiology unit, University of Torino, Via Genova 3, 10126 Turin, Italy; 2 Department of Surgical Sciences, Division of Vascular Surgery, University of Torino, Turin, Italy

**Keywords:** Subclavian artery injuries, Central venous catheterisation, Endovascular repair, Vascular closure device, Perclose Proglide

## Abstract

**Purpose:**

To present our experience and provide a literature review dissertation about the use of a suture-mediated percutaneous closure device (Perclose Proglide -PP- Abbott Vascular Inc., Santa Clara, CA, USA) to achieve hemostasis for unintended subclavian arterial catheterization during central venous line placement.

**Materials & methods:**

Since October 2020, we have successfully treated four consecutive patients with a central venous catheter (8 to 12 French) in the subclavian artery. In each patient, we released a PP, monitoring its efficacy by performing a subclavian angiogram and placing, as a rescue strategy, an 8 mm balloon catheter near the entry point of the misplaced catheter. Primary outcome is technical and clinical success. Technical success is defined as absence of bleeding signs at completion angiography, while clinical success is a composite endpoint defined as absence of hematoma, hemoglobin loss at 12 and 24 h, and absence of procedure-related reintervention (due to vessel stenosis, pseudoaneurysm or distal embolization).

**Results:**

Technical success was obtained in 75% of cases. In one patient a mild extravasation was resolved after 3 min of balloon catheter inflation. No early complications were observed for all patients.

**Conclusions:**

PP showed a safe and effective therapeutic option in case of unintentional arterial cannulation. It can be considered as first-line strategy, as it does not preclude the possibility to use other endovascular approaches in case of vascular closure device failure.

## Introduction

Despite the improvement of ultrasound guided catheterization, the incidence of unintended arterial puncture is still estimated to range between 2% and 4.5%, and large-bore catheter cannulation in 0.1 – 0.5% of patients. (Lorenzo et al. 2020; Ezaru et al. 2009; Brass et al. 2015). Subclavian artery is more frequently involved compared to carotid artery (2.7% vs. 1% respectively). Manual compression is often ineffective to achieve hemostasis due to the anatomical artery position (Park et al. 2016). Open surgery is burdened by high invasiveness and important blood loss. Endovascular use of covered stent-graft is a valuable alternative to open surgery but presents the risk of vertebral artery occlusion. Recently, vascular closure devices (VCD) have been used to treat the damaged vessel. This study aims to present a single center experience with Perclose Proglide (PP) (Abbott Vascular, Santa Clara, USA).

## Materials & methods

Since October 2020, first approach to treat unintentional artery cannulations of the upper extremity was managed with PP. All patients were preventively studied with CT angiography to confirm the site of arterial puncture and its relationship with contiguous structures and arterial branches. A doppler US was also performed to exclude the presence of arteriovenous fistulas.

### Technical procedure

All the procedures are performed in hybrid room under local anesthesia. The upper chest, neck, and right groin or right radial area are draped in a sterile fashion. Primary vascular access was ultrasound-guided through the common femoral artery (Radifocus Introducer 7Fr, Terumo, Japan) or radial artery (Prelude Ideal 7 F Merit, USA).

A preliminary angiography is performed to localize central venous line entrance into the artery wall. An 8-mm diameter occlusion balloon catheter is then advanced close to the damaged artery for hemostasis in case of VCD failure. The venous catheter is withdrawn on a 0.035-inch guidewire (Radifocus M guidewire standard type, Terumo, Japan) and a PP is inserted and deployed.

**Fig. 1:**
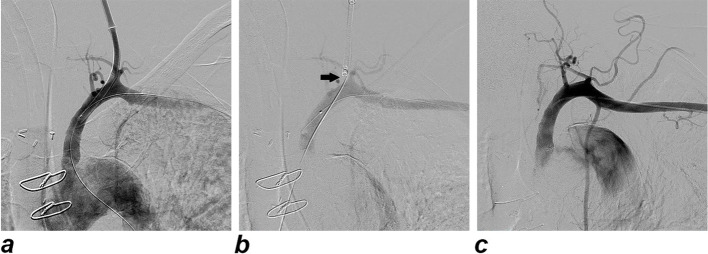
**a** Preliminary angiogram demonstrating the access site of the central venous catheter with the entry point located in between the vertebral artery and the thyrocervical trunk. An 8-mm diameter occlusion balloon catheter is placed in the subclavian artery near the entry point of the misplaced central venous catheter. **b** Angiogram executed after the deployment of the PP and during the tightening of the knot. On the upper side, the slipknot is located close to the arterial wall (arrow). **c** Final angiogram confirming the absence of active bleeding, stenosis or pseudoaneurysm

Completion angiography is performed to confirm the success of the procedure (Fig. 1). In case of persistent bleeding, a second device can be positioned, or, alternatively, a balloon inflated or stent graft deployed through the primary access.

### Clinical outcomes

Primary outcome is technical and clinical success. Technical success is defined as absence of bleeding signs at completion angiography, while clinical success is a composite endpoint defined as absence of hematoma, hemoglobin loss at 12 and 24 h, and absence of procedure-related reintervention (due to vessel stenosis, pseudoaneurysm or distal embolization).

Secondary outcomes are procedural time, fluoroscopy time, and amount of iodinate contrast medium used.

All patients were informed that the device used for hemostasis was not designed/tested for the specific purpose and gave their consent to treatment.

## Results

Four consecutive patients were treated for unintentional subclavian artery cannulation (3 males, average age 65.5 years). Main characteristics of each procedure are depicted in Table 1. Central venous catheter used ranged between 8 and 12 French caliber.

**Table 1:** Systematic overview of all patients

Patient ID	Age	Sex	Target vessel	Punctured vessel	Angiographic access	Technical success	Adjunctive treatment	Procedural time (minutes)	AK (mGy)	DAP (Gycm2)	Iodine Contrast medium (ml)
1	78	M	LJV	LS	LF (4 F)	Yes	No	35	1153	224	40
2	79	M	LJV	LS	LR (7 F)	No	Balloon insufflation	65	1000	127	50
3	47	M	RJV	RS	LF (7 F)	Yes	No	30	911	204	30
4	59	F	RSV	RS	LF (7 F)	Yes	No	25	1269	255	80

*LJV* left jugular vein, *RJV* right Jugular vein, *RSV* right subclavian vein

*LF* left femoral artery, *LR* left radial artery, *LS* left subclavian artery, *RS* right subclavian artery

Technical success was obtained in 75% of cases. Patient 2 presented a mild extravasation after PP deployment that resolved after 3 min of balloon catheter inflation. No early complications were observed for all patients. Mean procedural time was 39 ± 18 min, mean dose area product (DAP) was 203 ± 55 Gycm^2^, mean iodine contrast medium used was 50 ± 22 ml. Clinical success was achieved in all patients.

## Discussion

VCD has revolutionized percutaneous access hemostasis, offering a safe and effective alternative to manual compression (Makris et al. 2017). However, none of the VCDs has indication for subclavian artery.

Our limited experience with PP to achieve hemostasis in unintentional subclavian artery catheterization shows promising results with 100% of clinical success, high technical success, no need for stenting or surgical conversion, and with a small amount of iodinate contrast medium, procedural time and radiogenic exposure.

Similar experiences have been published in literature. Dornbos et al. (2019) reported 50 cases of VCD in both subclavian and brachiocephalic artery injuries with successful primary hemostasis in 94% of cases and no complications with PP. The 3 cases (6%) of failure requiring covered stenting were observed with the previous Perclose, Angioseal (St. Jude Medical, USA) and 1 unspecified VCD. 2 complications have been reported including formation of a pseudoaneurysm (StarClose, Abbott Vascular) (Stellmes et al. 2014) and vessel occlusion (Angioseal) (Sharma et al. 2008). Dornbos et al. (2019). Makris et al. (2017) reported that VCD appears to be safe and effective for the management of iatrogenic thoraco-cervical vascular injuries.

In our opinion, the advantage of PP device is double: the hemostasis is achieved with exclusively extravasal material and, in case of initial failure, the presence of the guide wire allows a second device deployment. Moreover, in case of PP failure, this technique does not preclude the use of a different endovascular approach (stent-graft).

Another advantage of VCD compared to the use of stent graft or surgical repair is the cost-effectiveness; a PP device is 10 times less expensive than a stent graft and costs of surgical hemostasis can be considered even higher.

To our knowledge, 10 cases of accidental subclavian artery puncture resolved with the placement of a PP have been reported (Table 2). In all cases, full technical success was achieved (with no need for stenting or surgical revision) without postoperative complications.

**Table 2:** Clinical studies reporting the use of Perclose Proglide device for the treatment of iatrogenic subclavian injuries

Author	Year	Number of Patients	Catheter size	Angiographic control	Technical success	Sequelae
Jahromi et al. (2009)	2009	1	8 F	Yes	Yes, balloon tamponade	No
Park et al. (2016)	2016	1	11.5	Yes	Yes	No
Yoon et al. (2015)	2015	2	7 and 9 F	Yes	Yes	No
Chivate et al. (2016)	2016	1	7 F	Yes	Yes	No
Lorenzo et al. (2020)	2020	5	7 F	No	Yes	No
Our experience	2021	4	8-12 F	Yes	Yes, one balloon tamponade	No

Although the PP has reduced profiles compared to the previous Perclose or the Prostar XL, the depth of the puncture site makes the descent of the suture thread rather difficult, even with the knot pusher (Berti et al. 2020). Due to the difficult positioning, the possibility of device failure and/or complications remains potentially high: We believe that the safest endovascular approach is possible when, prior to the deployment of a VCD, a balloon catheter is positioned on the entry site of the artery to tamponade eventual lesser vessel injury after VCD deployment, and to use it as a pre-existent way for exchange in case it is needed to place a covered stent. Although Lorenzo et al. (2020), we believe that bedside ultrasound-guided placement of PP should be limited to extremely critical and non-transportable patients.

## Conclusions

PP showed a safe and effective therapeutic option in case of unintentional arterial cannulation. It can be considered as a first-line strategy, as it does not preclude the possibility to use other endovascular approaches in case of VCD failure.

## Data Availability

The datasets used and/or analyzed during the current study are available from the corresponding author on reasonable request.

## Abbreviations

PP: Perclose Proglide
VCD: Vascular closure devices
